# Involvement of CX3CR1^+^ cells appearing in the abdominal cavity in the immunosuppressive environment immediately after gastric cancer surgery

**DOI:** 10.1186/s12957-024-03353-1

**Published:** 2024-03-04

**Authors:** Seiji Natsuki, Mami Yoshii, Hiroaki Tanaka, Takuya Mori, Sota Deguchi, Yuichiro Miki, Tatsuro Tamura, Takahiro Toyokawa, Shigeru Lee, Kiyoshi Maeda

**Affiliations:** 1 Department of Gastroenterological Surgery, Osaka City University Graduate School of Medicine, Asahi-machi 1-4-3, Abeno-ku, Osaka, Japan; 2 Department of Surgery, Fuchu Hospital, Osaka, Japan

**Keywords:** CX3CR1, Fractalkine receptor, Tumor-associated macrophage, Myeloid-derived suppressor cell, Programmed death 1, Intraperitoneal lavage fluid, Gastric cancer

## Abstract

**Background:**

Gastric cancer is primarily treated by surgery; however, little is known about the changes in the intraperitoneal immune environment and the prognostic impact of surgery. Surgical stress and cancer-associated inflammation cause immune cells to mobilize into the abdominal cavity via numerous cytokines. One such cytokine, CX3CR1, has various immune-related functions that remain to be fully explained. We characterized the intraperitoneal immune environment by investigating CX3CR1^+^ cells in intraperitoneal lavage fluid during gastric cancer surgery.

**Methods:**

Lavage fluid samples were obtained from a total of 41 patients who underwent gastrectomy. The relative expression of various genes was analyzed using quantitative real-time PCR. The association of each gene expression with clinicopathological features and surgical outcomes was examined. The fraction of CX3CR1^+^ cells was analyzed by flow cytometry. Cytokine profiles in lavage fluid samples were investigated using a cytometric beads array.

**Results:**

CX3CR1^high^ patients exhibited higher levels of perioperative inflammation in blood tests and more recurrences than CX3CR1^low^ patients. CX3CR1^high^ patients tended to exhibit higher pathological T and N stage than CX3CR1^low^ patients. CX3CR1 was primarily expressed on myeloid-derived suppressor cells and tumor-associated macrophages. In particular, polymorphonuclear myeloid-derived suppressor cells were associated with perioperative inflammation, pathological N, and recurrences. These immunosuppressive cells were associated with a trend toward unfavorable prognosis. Moreover, CX3CR1 expression was correlated with programmed death–1 expression.

**Conclusions:**

Our results suggest that CX3CR1^+^ cells are associated with an acute inflammatory response, tumor-promotion, and recurrence. CX3CR1 expression could be taken advantage of as a beneficial therapeutic target for improving immunosuppressive state in the future. In addition, analysis of intra-abdominal CX3CR1^+^ cells could be useful for characterizing the immune environment after gastric cancer surgery.

**Supplementary Information:**

The online version contains supplementary material available at 10.1186/s12957-024-03353-1.

## Introduction

Gastric cancer is treated using a variety of strategies, including chemotherapy and immune therapy; however, surgery is a primary mode of treatment. Surgical treatment for gastric cancer is both difficult and highly invasive for patients.

The prognosis for patients who develop complications after gastric cancer surgery is often poor. One reason for poor prognosis is that residual cancer cells may proliferate due to a decline in host immunocompetence. We previously investigated the intra-peritoneal immune environment using intraperitoneal lavage fluid (ILF) from patients with gastric cancer and demonstrated that the number of inflammatory cells within the ILF differed according to the type of operative approach [1]. This suggested an increase in intraperitoneal granulocytes occurs during the invasive surgical procedure to treat gastric cancer. However, little is known about the changes in the intraperitoneal immune environment that occur due to surgery and their prognostic impact.

Inflammatory cells such as macrophages migrate, infiltrate, and accumulate under the influence of various cytokines and chemokines. One such chemokine, CX3CR1, which is a fractalkine receptor for CX3CL1 [2], reportedly exhibits multiple functions [3]. CX3CR1 is expressed by immune cells such as natural killer cells, lymphocytes, macrophages, and myeloid-derived suppressor cells (MDSCs) [4]. When CX3CR1 binds to CX3CL1, multiple pathways are activated that cause tumor and immune cells to migrate, invade, and metastasize, ultimately leading to angiogenesis [3, 5, 6]. In various types of cancer, including gastric cancer, reports suggest that CX3CR1 is a poor prognostic factor that promotes metastasis [3, 7]. In contrast, some studies have reported an association between CX3CR1^+^ lymphocytes and the effectiveness of immune checkpoint inhibitors [8–10]. We therefore investigated the characteristics and prognostic impact of CX3CR1^+^ cells in ILF from patients with gastric cancer.

## Methods

### Patients and Samples

This study included 41 patients with gastric cancer who underwent surgical operation that enabled us to collect ILF at Osaka City University (currently Osaka Metropolitan University, Osaka, Japan) between January 2020 and June 2021. Depending on the tumor location, the surgeon performed proximal, distal, or total gastrectomy with lymphadenectomy. Attending surgeons selected patients to undergo either laparoscopic (and robot-assisted) or open surgery. Regarding clinicopathological findings, the histological types were determined based on differentiated type (well differentiated/moderately differentiated)/undifferentiated (poorly differentiated). All pathological stages were recorded according to the TNM Classification of Gastric Cancer, 8^th^ edition, published by the Union for International Cancer Control [11].

At the time of abdominal opening (designated ‘Opening’) or just before closing the abdominal wall (designated ‘Closure’), the abdominal cavity was irrigated using 500 mL of normal saline, and approximately 50 mL of lavage fluid was collected from the Douglas’ pouch. The obtained fluid was centrifuged at 300 × *g* for 10 min to pellet the cells. The cells were then resuspended in 1 mL of CELL BANKER (Nihon Zenyaku Kogyo Co. /Ltd.; cat. no. CB011) and stored at −80°C until further analysis.

This study was conducted according to the principles of the Declaration of Helsinki. All experimental procedures and follow-up were approved by the Osaka City University Ethics Committee (approval nos. 3138 and 4092). All patients provided written informed consent before enrollment.

### Quantitative real-time PCR

Quantitative real-time PCR was conducted to analyze the relative expression of various genes in ILF. Total RNA was isolated from cryopreserved cells using an RNeasy Mini kit (QIAGEN, Hilden, Germany), and the RNA was used as a template for synthesis of complimentary DNA by reverse transcription using a High-Capacity cDNA Reverse Transcription kit (Invitrogen, Carlsbad, CA, USA) in accordance with the manufacturer’s instructions. We performed quantitative real-time PCR using the Taq-Man Gene Expression Assay (Thermo Fisher Scientific, Waltham, MA, USA). Other than *CX3CR1* (Assay ID: Hs01922583_s1), MDSCs and tumor-associated macrophages (TAMs) were examined by assaying the relative expression of *OLR1* (LOX-1; lectin-like oxidized low-density lipoprotein receptor 1, Assay ID: Hs01552593_m1) and *CD163* (Assay ID: Hs00174705_m1). The relative expression of *PDCD1* (PD-1; programmed death 1, Assay ID: Hs01550088_m1) was also examined for determining the exhausted status of immune cells and immune checkpoint molecules. Thermocycling was performed using a 7500 Fast Real-Time PCR System (Applied Biosystems, Foster City, CA, USA) as follows: initial incubation at 95°C for 10 min, followed by 40 cycles of 95°C for 15 s and 60°C for 1 min. The δδCt method was used to quantitate and analyze the expression of *CX3CR1*, *OLR1*, *CD163*, and *PDCD1* relative to *GAPDH* as a control. The lowest expressed sample of each gene was designated the baseline sample to calculate the fold-change value. Receiver operating characteristic (ROC) curves were generated to determine cut-off values of gene expression, and patients were divided into two groups according to these values. ROC curves were based on 24-month relapse-free survival (RFS) for cut-off values of *CX3CR1*, *OLR1*, and *CD163* expression and 24-month overall survival (OS) for cut-off values of *PDCD1* expression (Supplementary Fig. 1).

### Flow cytometry

Flow cytometry was performed to determine the phenotypes of CX3CR1^+^ cells in ILF. Additional ILF samples (*n*=8) were collected by the same method for flow cytometry analyses. Red blood cells were lysed using RBC lysis buffer (eBioscience, San Diego, CA, USA), and the samples were washed with phosphate-buffered saline. Cells were subsequently saturated using BD Fetal Bovine Serum Stain Buffer (BD Biosciences) and incubated at 4°C for 30 min with the following antibodies to identify surface markers: BV421-labeled anti-CX3CR1 antibody (clone: 2A9-1: cat. no. 565800; BD biosciences). FITC-labeled anti-CD3 antibody (clone: UCHT1: cat. no. 555332; BD Biosciences) to detect T cells, and Alexa Fluor 488- labeled anti-FoxP3 antibody (clone: 25gD/C7: cat. no. 3270739; BD Biosciences) for regulatory T cells (Tregs). TAMs and monocytic MDSCs (M-MDSCs) were identified using PE-labeled anti-CD163 antibody (clone: GHI/61: cat. no. 556018; BD Biosciences) and BUV395-labeled anti-CD14 antibody (clone: MϕP9: cat. no. 563561; BD Biosciences). APC-labeled anti-LOX-1 antibody (clone: 15C4; cat. no. 358606; BioLegend) was primarily used to detect polymorphonuclear MDSCs (PMN-MDSCs). Dead cells were identified by staining with Fixable Viability Stain 780 (cat. no. 565388: BD Biosciences). Cells were permeabilized and fixed on ice for 20 min using Fixation/ Permeabilization Solution (BD Biosciences), and then they were washed with Perm/ Wash buffer (BD Biosciences) at 4°C for 30 min. A total of 3×10^4^ events were collected using a BD LSRFortessa^TM^ X-20 flow cytometer (BD Biosciences) with FACSDiva software (BD Biosciences). The resulting data were analyzed using FlowJo software, version 10 (Tree Star, Inc., Ashland, OR).

### Cytometric bead array

To quantify multiple cytokines in ILF, we used the Cytometric Bead Array (cat. no. 558264, BD Biosciences), a flow cytometry application that enables the simultaneous measurement of the concentrations of multiple proteins in a small sample volume. We performed the procedure as per the manufacturer’s instructions. GM-CSF (cat. no. 558335), IFN-γ (cat. no. 558269), IL-4 (cat. no. 558272), IL-6 (cat. no. 558276), IL-10 (cat. no. 558274), IL-12p70 (cat. no. 558283), MCP-1 (cat. no. 558287), MIP-1α cat. no. 558325), TNF (cat. no. 558273), and VFGF (cat. no. 558336) were analyzed, as these proteins are commonly secreted by MDSCs and macrophages. All of the cytokine flexes were obtained from BD Biosciences. We analyzed 25 samples that contained confirmable and sufficient proteins using Closure samples. A total of 3×10^3^ events were collected using a BD LSRFortessa^TM^ X-20 flow cytometer (BD Biosciences) with FACSDiva software (BD Biosciences). Protein concentrations were determined using BD CBA Analysis Software, version 1.0.2 (BD Biosciences). Protein levels were compared between CX3CR1^high^ and CX3CR1^low^ samples using the cut-off value for CX3CR1.

### Laboratory data

Laboratory data for before and after surgery were analyzed to identify any association with CX3CR1 expression. With regard to post-operative blood counts, complications other than surgical stress or cancer-associated inflammation, such as infection, can elevate inflammatory parameters. We then recorded the data on post-operative day (POD) 3, because systemic inflammation associated with surgical stress usually appears from POD 1 to 7, as we previously reported [1]. The neutrophil-to-lymphocyte ratio (NLR) and platelet-to-lymphocyte ratio (PLR) were determined at the time of admission for surgery and on POD 3.

### Statistical analysis

All statistical analyses were conducted using EZR [12], which is R with a modified version of R commander designed to add statistical functions frequently used in biostatistics. Fisher’s exact probability test was used to compare categorical variables. Continuous variables were compared using the Mann-Whitney *U* test. OS and RFS curves were compared using the Kaplan-Meier method, and the significance of differences in survival was assessed using the log-rank test. The date of surgery was set as the starting point for the measurement of OS and RFS. OS and RFS were defined based on the time of death and the time of death or recurrence, respectively. The correlogram and heatmap were generated using the web-based FaDA tool [13]. The correlogram was generated based on Spearman’s rank correlation coefficient. Hierarchical clustering in the heatmap was calculated using the average linkage method. *P*-values <0.05 were considered to indicate statistically significant differences.

## Results

### Association between CX3CR1, LOX-1, CD163, and PD-1 expression and clinical data before and after surgery

We analyzed the clinicopathological features of 41 patients with gastric cancer (Table 1). The mean age of the patients was 72 years (range, 43-89 years). The group included 31 men (75.6%) and 10 women (24.4%). The mean observation time was 18.2 months (range, 3-36 months).

**Table 1:** Clinicopathological characteristics (*n*=41)

Variables	N or median (range)	Variables		N or median (range)
Age [years old]	72 (43-89)	Hisological type		
Sex		differentiated/ undifferentiated adenoca.		22/ 19
Male/ Female	31/10	Tumor Location^b^		
Body Mass Index [kg/m2]	21.97 (16.59-33.58)	E/ U/ M/ L/ Other		3/ 8/ 11/ 18/ 1
Other cancer history	14 (34.1%)	pT category^a^		
cT-category^a^		1-2/ 3-4		16/ 25
1-2/ 3-4	13/ 28	pN-category^a^		
cN-category^a^		0/ 1-3		19/ 22
0/ 1-3	20/ 21	pStage^a^		
cStage^a^		I-II/ III-IV		26/ 15
I-II/ III-IV	21/ 20	Ly	present/ absent	18/ 23
Comorbidities (present, %)	35 (85.4%)	Vsss	present/ absent	17/ 24
Diabetes Mellitus	14 (34.1%)	Surgical operation		
Cardiac disease	3 (7.31%)	Open/ Laparoscopy		31/ 10
Renal disorder	1 (2.44%)	TG/ DG+PG^b^		14/ 27
Pulmonary disorder	6 (14.6%)	Lymph nodes dissection (D1/ 1+/ 2)		5/ 11/ 25
Hepatic disorder	6 (14.6%)	Bleeding [mL]		210 (5-1700)
		Operation time [min.]		259 (106-607)
		Anesthesia time [min.]		294 (148-640)
		Duration of hospitalization [days]		13 (8-64)

^a^TNM Classification of Gastric cancer, 8th ed

^b^ *Abbreviation*: *TG* Total gastrectomy, *DG* Distal gastrectomy, *PG* Proximal gastrectomy

*E* Esophagogastoric, *U* Upper third gastric, *M* Middle third gastric, *L* Lower third gastric

We then examined the relative expression of CX3CR1 using Opening and Closure samples by quantitative RT-PCR. Based on the determined cut-off value, patients were divided into two groups (CX3CR1^high^ or CX3CR1^low^). We also analyzed the association between CX3CR1 expression and various clinicopathological findings and operative outcomes (Table 2). In Opening samples, we did not find any association between these findings and CX3CR1 levels. In contrast, Closure samples exhibited significant differences between CX3CR1^high^ and CX3CR1^low^ patients. Although clinical Stage and patients’ comorbidities did not differ statistically in CX3CR1 levels, analysis of perioperative laboratory data indicated that CX3CR1^high^ patients showed higher NLR, PLR, and C-reactive protein (CRP) levels than CX3CR1^low^ patients. Moreover, there was a trend toward higher pathological T and N stage in the CX3CR1^high^ group than the CX3CR1^low^ group, though the difference was not statistically significant. In terms of operative outcomes, CX3CR1 expression was not affected by operative procedure or postoperative complications. Following clinical and pathological staging, CX3CR1^high^ patients underwent more extensive lymphadenectomy and received more adjuvant chemotherapy than CX3CR1^low^ patients without statistical differences. Also, the duration of hospitalization and recurrences after surgery differed significantly between the CX3CR1^high^ and CX3CR1^low^ groups.

**Table 2:** Association of CX3CR1 expression in intraperitoneal lavage fluid with clinicopathological data and operative outcomes

		CX3CR1					
		Opening			Closure		
		low (*n* = 31 )	high (*n* = 10)	*P*†	low (*n* = 17)	high (*n* = 24)	*P*†
Sex	Male/ Female	24/ 7	7/ 3	0.683	11/ 6	20/ 4	0.270
Age(years-old)		72.0 (69.5-76.0)	69.5 (65.3-77.3)	0.438	69.6 (65.0-73.0)	73.3 (69.0-78.3)	0.295
Tumor size (mm)		45.0 (30.0-80.5)	42.5 (23.0-54.5)	0.523	38.0 (30.0-50.0)	50.0 (33.8-86.3)	0.076
Comorbidities	absent/ present	4/ 27	2/ 8	0.622	3/ 14	3/ 21	0.679
Other cancer history	absent/ present	18/ 13	9/ 1	0.123	12/ 5	15/ 9	0.742
Preoperative NLR		2.66 (1.92-3.39)	1.98 (1.70-3.05)	0.235	1.79 (1.64-3.29)	2.75 (2.19-3.49)	0.037*
Postoperative NLR		6.65 (4.47-9.01)	6.65 (5.44-9.32)	0.574	5.56 (3.81-6.65)	7.38 (5.72-11.7)	0.005*
Preoperative PLR		113 (70.1-137)	78.1 (56.4-104)	0.118	56.0 (51.2-101)	124 (90.2-165)	<0.001*
Postoperative PLR		177 (128-236)	183 (97.1-261)	0.777	99.3 (79.0-174)	191 (175-491)	<0.001*
CRP POD3		12.1 (4.34-19.6)	12.2 (9.92-21.3)	0.649	6.29 (3.24-14.3)	14.2 (10.1-23.8)	0.010*
cT stage^a^	T1-2/ 3-4	9/ 22	4/ 6	0.698	7/ 10	6/ 18	0.322
cN stage^a^	N0/ 1-3	15/ 16	5/ 5	1	9/ 8	11/ 13	1
cStage^a^	Stage I-II/ III-IV	16/ 15	5/ 5	1	8/ 9	13/ 11	0.756
Histological type	differentiated/ undifferentiated	18/ 13	4/ 6	0.469	10/ 7	12/ 12	0.752
pT stage^a^	T1-2/ 3-4	10/ 21	6/ 4	0.15	10/ 7	6/ 18	0.051
pN number		2.00 (0.00-3.00)	0.00 (0.00-1.75)	0.361	0.00 (0.00-3.00)	2.00 (0.00-3.00)	0.194
pN stage^a^	N0/ 1-3	13/ 18	6/ 4	0.469	11/ 6	8/ 16	0.062
pStage^a^	Stage I-II/ III-IV	20/ 11	7/ 3	1	13/ 4	14/ 10	0.321
Lymphatic invasion	absent/ present	16/ 15	7/ 3	0.467	12/ 5	11/ 13	0.201
Venous invasion	absent/ present	17/ 14	7/ 3	0.48	12/ 5	12/ 12	0.217
Operative approach	open/ laparoscopic	25/ 6	6/ 4	0.222	12/ 5	19/ 5	0.714
Operative procedure	DG or PG/ TG	19/ 12	8/ 2	0.447	13/ 4	14/ 10	0.321
Lymphadenectomy	D1 or 1+/ D2	10/ 21	6/ 4	0.150	10/ 7	6/ 18	0.051
Operative time (min)		251 (213-289)	300 (239-324)	0.092	236 (219-306)	261 (225-305)	0.634
Anesthesia time (min)		289 (246-322)	314 (275-369)	0.141	277 (265-331)	296 (264-339)	0.525
Bleeding (mL)		200 (50-380)	440 (35-844)	0.370	80 (20-510)	265 (73-410)	0.283
Comlplications	absent/ present	20/ 11	6/ 4	1	12/ 5	14/ 10	0.519
Duration of hospitalization (days)		13.0 (10-18.5)	12.0 (11-18.3)	0.867	11.0 (9-14)	13.5 (11-21)	0.039*
Adjuvant chemotherapy	absent/ present	13/ 18	6/ 4	0.469	11/ 6	8/ 16	0.062
Recurrence	absent/ present	23/ 8	5/ 5	0.241	15/ 2	13/ 11	0.039*
Recurrent style (local/ lymphatic/ hematogenous/ dissemination)		0/ 2/ 3/ 5	1/ 0/ 1/ 2	0.566	0/ 0/ 0/ 2	1/ 2/ 4/ 5	0.692

Data are expressed as median (interquarantile range) or the number

*NLR* Neutrophil-to-lymphocyte ratio, *PLR* Platelet-to-lymphocyte ratio, *POD* Post-operative day, *DG* Distal gastrectomy, *PG* Proximal gastrectomy, *TG* Total gastrectomy

^†^Fisher's exact probability test to categorical variables, Mann Whitney *U* test to continuous variables. *p*<0.05, statistically significant

^a^UICC-TNM Classification of Gastric Cancer, 8th ed

**Fig. 1:**
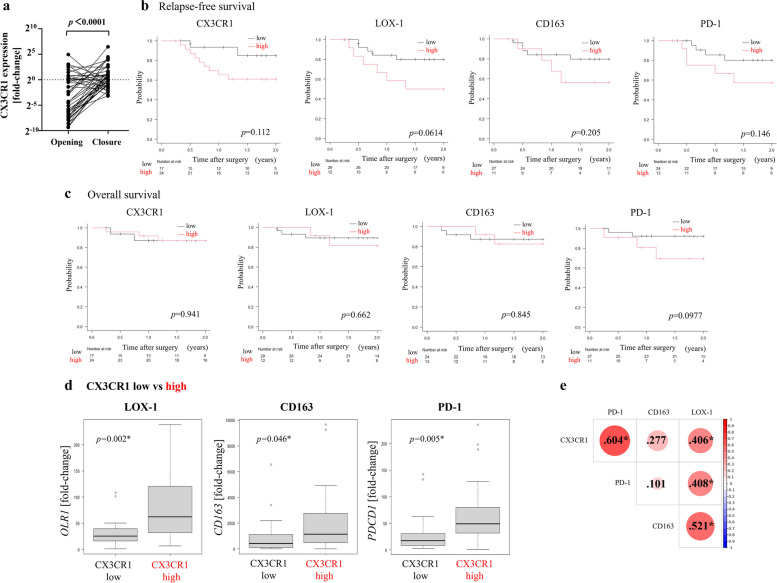
**a** The change of CX3CR1 expression through surgical maneuvers. **b**, **c** Comparison of relapse-free survival and overall survival based on levels of various molecules. *P*<0.05, statistically significant. **b** Relapse-free survival. **c** Overall survival. **d** Comparison of *OLR1*, *CD163*, and *PDCD1* gene expression between CX3CR1^high^ and CX3CR1^low^ patients. *P<0.05, statistically significant. **e** Correlogram. Circle size indicates the degree of correlation. Figures in circles indicate correlation coefficients. Darker colors of circles indicate more-significant correlations. *P<0.05, statistically significant

We analyzed the change of CX3CR1 expression in ILF before and after surgical maneuvers designating the cut-off sample as a baseline (Fig .1a). As a result, we found that CX3CR1 expression in a lot of ILF elevated through surgery (*p*<0.0001). Based on CX3CR1^high^ or CX3CR1^low^ at ‘Opening’ and ‘Closure’, we designated CX3CR1 changing groups as high-high, low-high, high-low, and low-low (Opening-Closure). We then analyzed their association with clinicopathological features and operative outcomes (Table 3). The low-high group showed relatively high preoperative inflammatory markers, whereas the high-low group had as low levels of those as the low-low group. The low-high group also had a trend toward higher pT stages and more recurrences, and underwent more extensive lymphadenectomy than CX3CR1^low^ groups at ‘Closure’.We also examined LOX-1 (*OLR1* gene) and CD163 (*CD163* gene) expression in Closure ILF using quantitative real-time PCR. In addition, to identify immunosuppressive status and immune cell exhaustion, PD-1 (*PDCD1* gene) expression was investigated. In Closure samples, sufficient levels of LOX-1, CD163, and PD-1 were obtained from 41, 37, and 38 patients, respectively. We evaluated whether the levels of LOX-1, CD163, and PD-1 had any effect on clinicopathological features and operative outcomes (Table 4). Patients were divided into two groups based on the aforementioned cut-off values (Supplementary Fig. 1). Neither CD163 nor PD-1 expression was significantly associated with clinicopathological findings or blood test parameters. However, the LOX-1^high^ group had higher perioperative NLR and PLR, more extensive lymph node metastasis, and more extensive vascular invasion than the LOX-1^low^ group. No significant differences were observed between perioperative outcomes and LOX-1, CD163, and PD-1 expression. LOX-1 expression was associated with postoperative recurrences significantly.

**Table 3:** Association of CX3CR1 dynamic change in intraperitoneal lavage fluid with clinicopathological data and operative outcomes

		CX3CR1 change 'Opening' to 'Closure'				
		high-high (*n*=5)	low-high (*n*=19)	high-low (*n*=5)	low-low (*n*=12)	*P*†
Sex	Male/ Female	1/ 4	3/ 16	2/ 3	4/ 8	0.573
Age(years-old)		75.0 (67.0-78.0)	72.0 (69.5-77.5)	65.0 (57.0-72.0)	71.5 (70.3-73.8)	0.542
Tumor size (mm)		56.0 (45.0-70.0)	50.0 (32.5-87.5)	38.0 (18.0-40.0)	37.5 (30.0-56.3)	0.258
Comorbidities	absent/ present	1/ 4	2/ 17	1/ 4	2/ 10	0.913
Other cancer history	absent/ present	5/ 0	10/ 9	4/ 1	8/ 4	0.211
Preoperative NLR		2.20 (2.16-3.34)	2.85 (2.28-3.52)	1.69 (1.58-1.72)	2.14 (1.75-3.30)	0.123
Postoperative NLR		7.43 (5.41-25.7)	7.32 (5.83-10.1)	5.88 (5.56-8.44)	4.13 (3.54-6.14)	0.025*
Preoperative PLR		111 (80.4-131)	125 (93.7-178)	53.5 (40.6-65.0)	64.0 (51.2-115)	0.004*
Postoperative PLR		186 (182-560)	195 (173-399)	89.7 (79.0-186)	113 (83.8-167)	0.010*
CRP POD3		20.5 (10.1-23.2)	13.9 (9.84-23.9)	9.90 (8.35-14.3)	4.34 (2.94-3.0)	0.065
cT stage^a^	T1-2/ 3-4	2/ 3	4/ 15	2/ 3	5/ 7	0.601
cN stage^a^	N0/ 1-3	3/ 2	15/ 4	4/ 1	9/ 3	0.842
cStage^a^	Stage I-II/ III-IV	2/ 3	11/ 8	3/ 2	5/ 7	0.757
Histological type	differentiated/ undifferentiated	1/ 4	11/ 8	3/ 2	7/ 5	0.457
pT stage^a^	T1-2/ 3-4	2/ 3	4/ 15	4/ 1	6/ 6	0.082
pN number		1.0 (0.0-2.0)	2.0 (0.0-3.0)	0.0 (0.0-0.0)	0.0 (0.0-3.25)	0.498
pN stage^a^	N0/ 1-3	4/ 1	10/ 9	4/ 1	8/ 4	0.533
pStage^a^	Stage I-II/ III-IV	3/ 2	11/ 8	4/ 1	9/ 3	0.682
Lymphatic invasion	absent/ present	4/ 1	7/ 12	3/ 2	9/ 3	0.122
Venous invasion	absent/ present	3/ 2	9/ 10	4/ 1	8/ 4	0.521
Operative approach	open/ laparoscopic	2/ 3	3/ 16	2/ 3	3/ 9	0.555
Operative procedure	DG or PG/ TG	4/ 1	10/ 9	4/ 1	9/ 3	0.421
Lymphadenectomy	D1 or 1+/ D2	3/ 2	3/ 16	3/ 2	7/ 5	0.045*
Operative time (min)		294 (261-322)	259 (213-286)	306 (230-325)	234 (211-283)	0.353
Anesthesia time (min)		321 (306-370)	294 (246-324)	339 (273-365)	291 (248-333)	0.469
Bleeding (mL)		370 (50.0-615)	230 (110-375)	510 (20.0-920)	70.0 (13.8-395)	0.434
Comlplications	absent/ present	3/ 2	11/ 8	3/ 2	9/ 3	0.803
Duration of hospitalization (days)		13 (11-20)	14 (11.5-21.5)	11 (11-13)	10 (9.0-14)	0.184
Adjuvant chemotherapy	absent/ present	2/ 3	6/ 13	4/ 1	7/ 5	0.194
Recurrence	absent/ present	2/ 3	12/ 7	4/ 1	11/ 1	0.135
Recurrent style (local/ lymphatic/ hematogenous/ dissemination)		1/ 0/ 1/ 1	0/ 2/ 3/ 4	0/ 0/ 0/ 1	0/ 0/ 0/ 1	0.771

Data are expressed as median (interquarantile range) or the number

*NLR* Neutrophil-to-lymphocyte ratio, *PLR* Platelet-to-lymphocyte ratio, *POD* Post-operative day, *DG* Distal gastrectomy, *PG* Proximal gastrectomy, *TG* Total gastrectomy

^†^Fisher's exact probability test to categorical variables, Mann Whitney *U* test to continuous variables. *p*<0.05, statistically significant

^a^UICC-TNM Classification of Gastric Cancer, 8th ed

**Table 4:** Relevance to clinicopathological characteristics and operative outcomes with LOX-1, CD163, and PD-1 expression

		LOX-1			CD163			PD-1		
		Closure			Closure			Closure		
		low (*n* = 29)	high (*n* = 12)	*P*†	low (*n* = 24)	high (*n* = 13)	*P*†	low (*n*=27)	high (*n*=11)	*P*†
Sex	Male/ Female	22/ 7	9/ 3	1	18/ 6	10/ 3	1	22/ 5	8/ 3	0.667
Age(years-old)		71.0 (65.0-73.0)	72.5 (69.0-78.3)	0.295	72.0 (69.8-76.8)	71.0 (67.0-73.0)	0.202	71.0 (67.5-72.5)	75.0 (72.0-79.0)	0.032*
Tumor size (mm)		40.0 (30.0-65.0)	50.0 (40.0-87.0)	0.240	40.0 (28.0-61.3)	45.0 (30.0-56.0)	0.556	40.0 (27.5-62.5)	45.0 (32.5-63.0)	0.640
Comorbidities	absent/ present	3/ 26	3/ 9	0.334	4/ 20	2/ 11	1	4/ 23	2/ 9	1
Other cancer history	absent/ present	20/ 9	7/ 5	0.719	16/ 8	8/ 5	1	17/ 10	8/ 3	0.714
Preoperative NLR		2.17 (1.79-2.97)	3.23 (2.54-4.45)	0.037*	2.17 (1.79-3.01)	3.12 (2.20-3.97)	0.124	2.36 (1.76-3.31)	2.24 (2.07-4.46)	0.376
Postoperative NLR		5.97 (4.28-8.33)	8.48 (5.33-23.3)	0.140	5.96 (4.53-8.54)	6.91 (4.66-15.9)	0.455	5.97 (4.32-8.63)	7.43 (4.95-16.6)	0.247
PreoperativePLR		78.0 (53.6-124)	132 (108-189)	0.003*	79.2 (54.2-128)	123 (92.9-132)	0.102	82.3 (53.9-129)	111 (86.6-131)	0.274
Postoperative PLR		164 (90.8-203)	201 (180-568)	0.018*	160 (90.5-207)	186 (177-560)	0.102	157 (90.2-252)	186 (173-407)	0.111
cT stage^a^	T1-2/ 3-4	11/ 18	2/ 10	0.276	10/ 14	2/ 11	0.149	10/ 17	3/ 8	0.714
cN stage^a^	N0/ 1-3	16/ 13	4/ 8	0.306	13/ 11	6/ 7	0.737	12/ 15	7/ 4	0.476
cStage^a^	Stage I-II/ III-IV	17/ 12	4/ 8	0.181	14/ 10	6/ 7	0.512	14/ 13	6/ 5	1
CRP POD3		11.5 (4.67-17.5)	14.2 (7.12-23.3)	0.547	11.37 (5.43-17.5)	12.1 (3.24-23.2)	0.987	12.2 (4.64-19.6)	12.1 (10.7-21.9)	0.394
Histological type	differentiated/ undifferentiated	14/ 15	8/ 4	0.325	12/ 12	7/ 6	1	15/ 12	5/ 6	0.724
pT stage^a^	T1-2/ 3-4	12/ 17	4/ 8	0.734	11/ 13	4/ 9	0.491	11/ 16	4/ 7	1
pN number		0.00 (0.00-3.00)	2.50 (1.75-3.25)	0.065	0.00 (0.00-4.25)	2.00 (0.00-2.00)	0.973	1.00 (0.00-3.00)	1.00 (0.00-4.00)	0.785
pN stage^a^	N0/ 1-3	17/ 12	2/ 10	0.019*	13/ 11	5/ 8	0.495	13/ 14	5/ 6	1
pStage^a^	Stage I-II/ III-IV	20/ 9	7/ 5	0.719	17/ 7	9/ 4	1	19/ 8	6/ 5	0.457
Lymphatic invasion	absent/ present	19/ 10	4/ 8	0.087	14/ 10	8/ 5	0.460	15/ 12	7/ 4	0.729
Venous invasion	absent/ present	20/ 9	4/ 8	0.045*	13/ 11	9/ 4	0.638	17/ 10	6/ 5	0.722
Operative approach	open/ laparoscopic	20/ 9	6/ 6	0.300	16/ 8	11/ 2	0.440	19/ 8	7/ 4	0.714
Operative procedure	DG or PG/ TG	20/ 9	7/ 5	0.719	19/ 5	6/ 7	0.067	7/ 20	2/ 9	1
Lymphadenectomy	D1 or 1+/ D2	14/ 15	2/ 10	0.084	9/ 15	6/ 7	0.730	10/ 17	6/ 5	0.471
Operative time (min)		251 (223-309)	264 (196-297)	0.764	243 (214-303)	268 (251-306)	0.382	236 (213-304)	267 (246-308)	0.385
Anesthesia time (min)		294 (268-352)	292 (233-315)	0.422	274 (250-333)	312 (279-335)	0.259	277 (250-322)	306 (282-344)	0.311
Bleeding (mL)		140 (20-380)	270 (142-651)	0.105	100 (20-373)	380 (150-750)	0.0502	210 (45-470)	150 (40-683)	0.735
Complications	absent/ present	20/ 9	6/ 6	0.300	17/ 7	8/ 5	0.716	17/ 10	7/ 4	1
Duration of hospitalization (days)		11.0 (10-18)	14.0 (13-20)	0.084	11.5 (10-14)	14 (11-20)	0.291	12.0 (10-18)	13.0 (11-23)	0.457
Adjuvant chemotherapy	absent/ present	16/ 13	3/ 9	0.098	14/ 10	4/ 9	0.17	11/ 16	7/ 4	0.288
Recurrence	absent/ present	24/ 5	5/ 7	0.020*	20/ 4	7/ 6	0.118	21/ 6	7/ 4	0.432
Recurrent style (local/ lymphatic/ hematogenous/ dissemination)		1/ 1/ 1/ 2	0/ 1/ 3/ 5	0.629	1/ 1/ 2/ 2	0/ 0/ 1/ 5	0.318	0/ 0/ 1/ 5	1/ 1/ 2/ 2	0.318

Data are expressed as median (interquarantile range) or the number

*NLR* Neutrophil-to-lymphocyte ratio, *PLR* Platelet-to-lymphocyte ratio, *POD* Post-operative day, *DG* Distal gastrectomy, *PG* Proximal gastrectomy, *TG* Total gastrectomy

^†^Fisher's probability exact test to categorical variables, Mann Whitney *U* test to continuous variables. *p*<0.05, statistically significant

^a^UICC-TNM Classification of Gastric Cancer, 8th ed

### Association between CX3CR1, LOX-1, CD163, and PD-1 expression and survival benefits

Prognostic impacts were examined based on the expression of CX3CR1, LOX-1, CD163, and PD-1 in ‘Closure’ ILF. (Fig. 1b, c). Although not statistically significant, all high-level CX3CR1, LOX-1, CD163, and PD-1 patients tended to have poorer RFS. With regard to OS, there were no significant differences between the high and low CX3CR1, LOX-1, CD163, and PD-1 expression groups.

### Relationship between CX3CR1^+^ cell phenotype and LOX-1 and CD163

We compared the expression of LOX-1, CD163, and PD-1 between the CX3CR1^high^ and CX3CR1^low^ groups in ‘Closure’ ILF. (Fig. 1d). The CX3CR1^high^ group had higher levels of all three molecules than the CX3CR1^low^ group. A correlogram for all three molecules is shown in Fig. 1e. CX3CR1 level was significantly correlated with PD-1 and LOX-1 expression. In particular, LOX-1 expression exhibited significant correlations with the expression of CX3CR1, CD163, and PD-1.

**Fig. 2:**
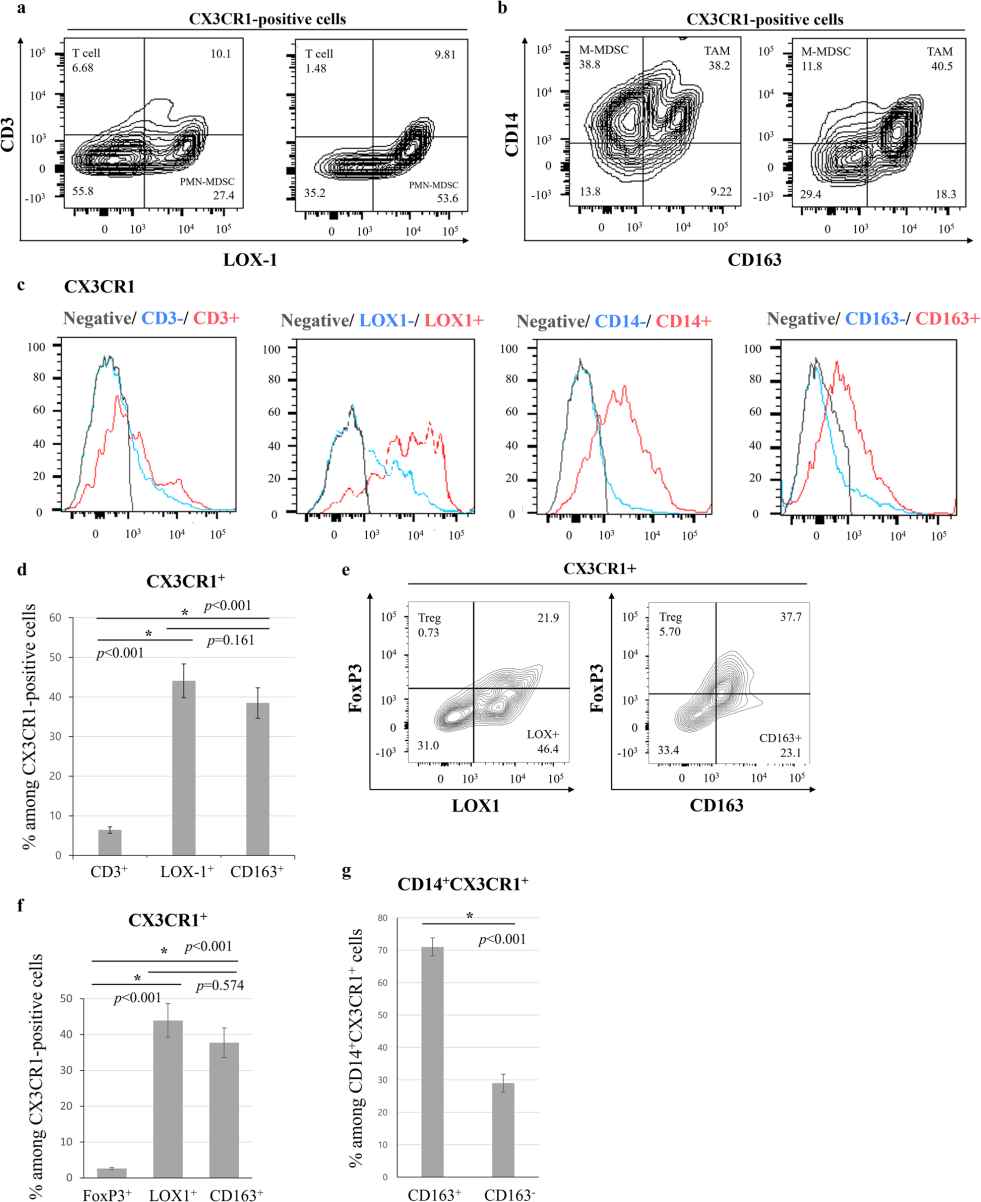
Flow cytometry analysis (*n*=8). **a** Representative contour plots of CD3 and LOX-1 positivity among CX3CR1^+^ cells. **b** Representative contour plots of CD14 and CD163 positivity among CX3CR1^+^ cells. **c** Representative histograms of CX3CR1^+^ cells classified by CD3, LOX-1, CD14, and CD163 positivity. **d**, **e** Bar graphs showing the mean and standard error. (**d**) Comparison of CD3, LOX-1, and CD163 positivity among CX3CR1^+^ cells. *P<0.05, statistically significant. NS, not significant. **e** Representative contour plots of FoxP3, LOX-1, and CD163 positivity among CX3CR1^+^ cells. **f** Comparison of FoxP3, LOX-1, and CD163 positivity among CX3CR1^+^ cells. *P<0.05, statistically significant. **g** Comparison of CD163 expression among CD14^+^CX3CR1^+^ cells. *P<0.05, statistically significant.

Considering the tumor-promoting trend and high inflammation parameters in Closure CX3CR1^high^ patients, we used flow cytometry to classify the phenotypes of CX3CR1^+^ cells. With regard to tumor-promoting or immunosuppressive cells, CX3CR1 is reportedly expressed by Tregs, MDSCs, and TAMs [3]; therefore, we analyzed CD3 expression in Tregs, CD14 and CD163 expression in M-MDSCs and TAMs, and LOX-1 expression in PMN-MDSCs. Representative contour plots are shown in Fig. 2a, b. Representative histograms of these molecules are shown in Fig. 2c and revealed that the presence of these molecules was associated with CX3CR1 expression. We then analyzed the proportion of CX3CR1^+^ cells in ILF (Fig. 2d). This analysis showed that CX3CR1^+^ cells rarely express CD3 (6%), whereas a large number of LOX-1^+^ PMN-MDSCs (44%) and CD163^+^ TAMs (38%) were present. We confirmed the Treg population using a FoxP3 antibody as shown in Fig. 2e and observed a similar trend to Fig. 2d (Fig. 2f; Tregs, 2.6%). With regard to the fraction of CD14^+^ cells among CX3CR1^+^ cells, CD163^+^ TAMs constituted approximately 70% of CD14^+^CX3CR1^+^ cells (Fig. 2g).

### Cytokine levels within ILF

**Fig. 3:**
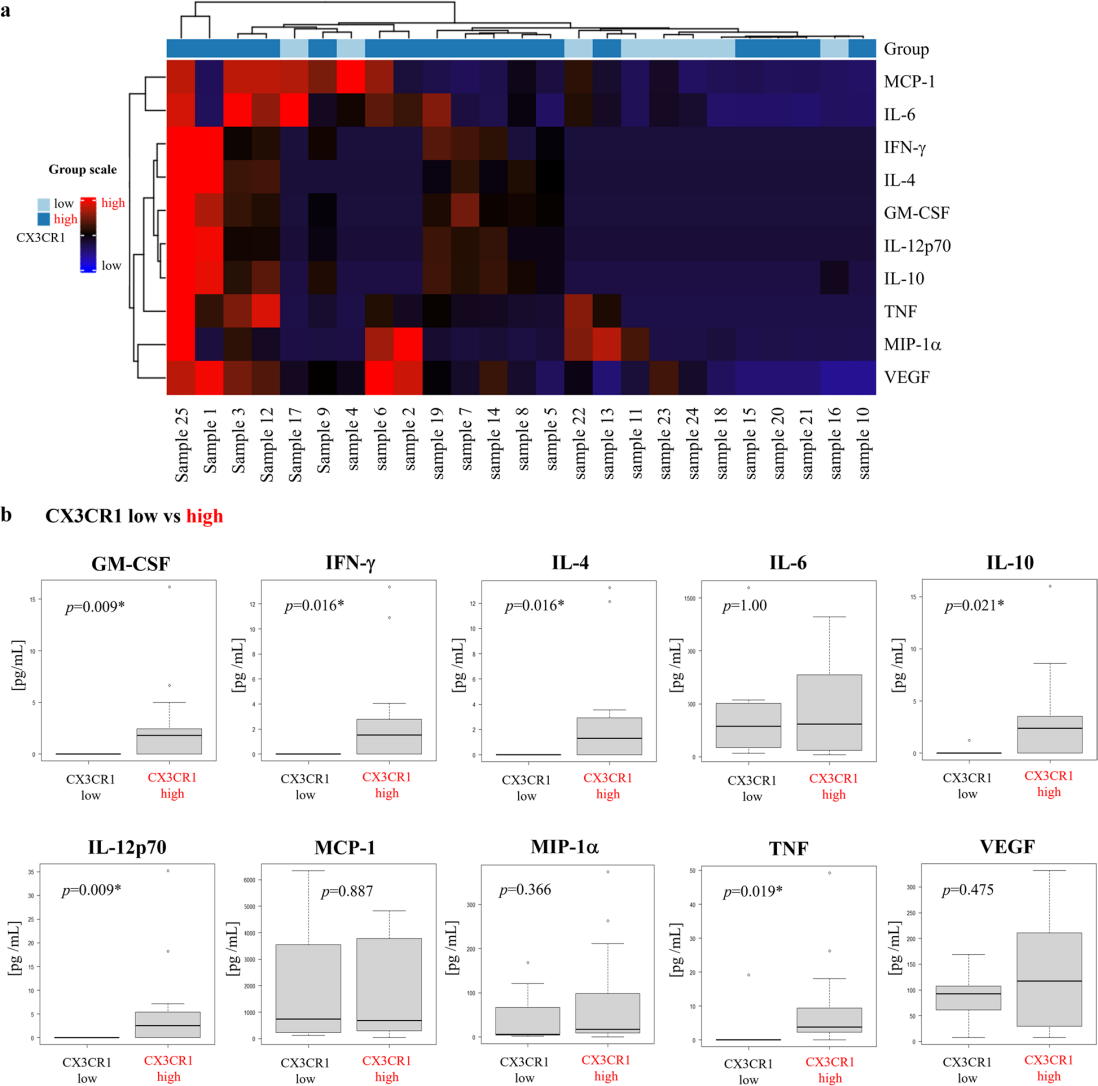
Cytokine profiles of Closure samples. **a** Heatmap. CX3CR1 group is divided into two subgroups: the high group is shown in dark blue, and the low group is shown in light blue. **b** Comparison of levels of various cytokines between the CX3CR1^high^ and CX3CR1^low^ groups. *P*<0.05, statistically significant.

We used some Closure samples to analyze cytokine profiles and characterize local immune environment parameters such as promotion of inflammation and macrophage polarization. A total of 25 Closure samples contained sufficient protein for analysis, consisting of 17 CX3CR1^high^ and 8 CX3CR1^low^ samples. A heatmap showing the expression of 10 cytokines in these 25 samples is shown in Fig. 3a. The data indicated that compared with CX3CR1^low^ samples, MDSCs and TAMs in CX3CR1^high^ samples tended to secrete higher levels of various cytokines, including IL-4, IL-10, GM-CSF, and IFN-γ. We then compared the cytokine concentrations in detail between CX3CR1^high^ and CX3CR1^low^ samples (Fig. 3b). Both groups exhibited elevated levels of inflammatory proteins such as IL-6, MCP-1, MIP-1α, and VEGF, but the differences were not statistically significant. In addition, some CX3CR1^high^ samples contained IL-4 and IL-10, whereas these proteins were rarely detected in CX3CR1^low^ samples. Levels of GM-CSF, IFN-γ, IL-12p70, and TNF were also significantly higher in the CX3CR1^high^ group than in the CX3CR1^low^ group.

## Discussion

The results of the present study suggest that CX3CR1^+^ cells appearing in the abdominal cavity after gastric cancer surgery are associated with an acute inflammatory response and that these cells may originate from immunosuppressive macrophages and MDSCs. Our data also suggest that the presence of CX3CR1^+^ cells affect long-term prognosis. According to previous reports, the CX3CR1-CX3CL1 axis regulates the migration, invasion, and metastasis of tumor and immune cells [3, 5, 6]. This axis interacts with and recruits TAMs and MDSCs in tumor sites [3]; therefore, these cells express CX3CR1 in tumors, as observed in our study. These immune suppressive cells would affect the difference in the trend of pathological T and N stage, and CX3CR1^high^ patients ended up receiving slightly more adjuvant chemotherapy and significantly relapsing more than CX3CR1^low^ patients as shown in Table 2. With regard to recurrent styles, although there was no statistical significance, CX3CR1 expression was associated with the ease of relapse at any place including dissemination. We speculated that CX3CR1^+^ cells that have already circulated in patients affect hematogenous and lymphatic metastasis and they then migrate in the abdominal cavity by surgical procedure getting tumor cells to escape antitumor immunity to disseminate. We examined the phenotypes of CX3CR1^+^ cells in ILF using flow cytometry. In addition to MDSCs and TAMs, CX3CR1^+^ immunosuppressive cells are also thought to include Tregs. However, our results showed that LOX-1^+^ PMN-MDSCs and CD163^+^ TAMs are major CX3CR1^+^ cells but that T lymphocytes such as Tregs are rarely present. In other words, our data suggest that CX3CR1^+^ PMN-MDSCs and TAMs in particular exhibit immunosuppressive effects in the intraperitoneal immune microenvironment.

Although we prepared both Opening and Closure samples, CX3CR1 expression in Closure samples was associated with various blood parameters and a tumor-promoting status. This result suggests that surgery promotes the invasion of CX3CR1^+^ cells within the peritoneum and increased blood flow. In the peritoneum and omentum, immune cells aggregate to form a structure like a secondary lymphoid organ [14]. Surgery-related stress and cancer-associated inflammation reportedly induce immune cells to mobilize in the peritoneal cavity from the peritoneum or the omental immune structure [14, 15]. In this study, CX3CR1 expression did not differ statistically in operative outcomes as shown in Table 2. Without statistical significance, there were trends that CX3CR1^high^ patients underwent more extensive lymphadenectomy and had more bleeding. In Table 3, the low-high group underwent more extensive lymphadenectomy than CX3CR1^low^ groups at ‘Closure’. Lymphadenectomy and surgical bleeding might be associated with CX3CR1^+^ cell migration into the abdominal cavity. Relationships between CX3CR1 cell migration, bleeding, and lymphadenectomy would be proven by a larger study. As shown in Tables 2 and 3, the present study suggests that collecting ILF immediately after a surgical procedure is valid for investigating the intraperitoneal immune environment.

We examined the association between CX3CR1 expression and various blood count parameters. Perioperative blood count parameters such as the NLR and PLR are reportedly poor prognostic factors in gastric cancer [16, 17]. The NLR is reportedly an indicator of tumor-associated neutrophils, thereby linking to the association with poor prognosis [18]. The NLR is also related to surgical stress. We previously reported that more-invasive surgery leads to the release of more cytokines and the mobilization of more granulocytes into the peritoneum and blood [1]. Platelets also play a role in the inflammatory response and the development of pro-tumoral inflammation [19]. In this study, the CX3CR1^high^ group exhibited elevated perioperative NLR and PLR. CX3CR1 expression was associated with not only postoperative parameters but also preoperative parameters, which were speculated to relate with cancer-associated inflammation caused by latent presence of many immunosuppressive cells. The proportion of LOX-1^+^ PMN-MDSCs among CX3CR1^+^ cells was also associated with NLR and PLR. Our results suggest that surgery-related stress and cancer-associated inflammation in particular are mediated by CX3CR1^+^ PMN-MDSCs. We also hypothesize that these cells are related to an unfavorable prognosis. As a promising monitor of acute inflammatory response, CX3CR1 expression may have advantages over NLR and PLR in predicting tumor-promotion status, possibility of unfavorable prognosis, and postoperative recurrence. We may be able to take advantage of CX3CR1^+^ immunosuppressive cells as a novel therapeutic target, unlike blood count parameters. CRP levels were highly elevated in the CX3CR1^high^ group. Elevation of these inflammatory markers thus probably plays a role in the longer hospital stay of CX3CR1^high^ patients after surgery than CX3CR1^low^ patients due to the need for treatment of inflammation. CX3CR1^+^ cells in patients’ blood might tell us their circulation, but their examination is somewhat invasive.

We found CX3CR1^+^ PMN-MDSCs and TAMs in ILF, but these tumor-promoting cells reportedly interact with each other, as MDSCs promote TAM differentiation [20, 21], and interaction can differentiate PMN-MDSCs and M-MDSCs [22]. In our study, MDSCs and TAMs constituted a majority of CX3CR1^+^ cells and probably exhibited a bidirectional interaction. We therefore examined the cytokine profiles in peritoneal lavage fluids. The CX3CR1^high^ group expressed higher levels of cytokines than the CX3CR1^low^ group. Similar levels of inflammation-promoting cytokines were detected in samples from both groups. However, significantly higher levels of GM-CSF, IFN-γ, IL-4, and IL-10 released by MDSCs and TAMs were detected in the CX3CR1^high^ group. These results support the hypothesis that a higher frequency of CX3CR1^+^ PMN-MDSCs and TAMs is associated with systemic tumor-promoting conditions. Although significantly higher levels of IL-12, which is typically released by M1-macrophages, were also detected in the CX3CR1^high^ group, we speculate that interactions between subtypes of macrophages played a role in this result.

Our results also call attention to the relationship between PD-1 expression and CX3CR1^+^ cells. Previous studies reported that CX3CR1^+^CD8^+^ T cells are important in antitumor immunity and associated with the response to PD-1 therapy [8, 9]. In this study, CX3CR1 was expressed on immunosuppressive cells rather than antitumor immune cells. However, immunosuppressive cells such as MDSCs and TAMs upregulate Tregs and release various cytokines leading to suppressing T cell activation, inducing to tumor-progression, and upregulating PD-L1 of the tumor: they finally promote PD-1 expression and antitumor cell exhaustion [22, 23]. Our correlogram also revealed that CX3CR1 expression is correlated with PD-1 expression. In other words, ILF could be used to determine the exhaustion status of immune cells within the intraperitoneal immune environment. Characterizing the intraperitoneal condition could enable clinicians to predict the effectiveness of immune checkpoint inhibitors such as anti–PD-1 antibodies. CX3CR1 may also be a prognostic predictor and novel treatment target.

This study has several limitations. First, the number of eligible patients was small, and the observation period was short, so bias was likely. A more-extensive and longer-term study with a larger sample size is required to reinforce our results. Second, as this study primarily utilized quantitative real-time PCR and flow cytometry, we could not elucidate functional and mechanistic details. Further studies are also necessary in this regard.

## Conclusions

In conclusion, CX3CR1 is expressed on immunosuppressive cells in ILF, including MDSCs and TAMs. These cells are associated with inflammatory parameters and tumor-promotion. CX3CR1^+^ cells could be taken advantage of as a novel therapeutic target in the future. In addition, analyses of the intraabdominal immune environment could be useful in the development of new therapeutic strategies to treat gastric cancer.

### Supplementary Information

**Supplementary Material 1.**

**Supplementary Material 2.**

## Data Availability

No datasets were generated or analysed during the current study.

## Abbreviations

ILF: intraperitoneal lavage fluid
MDSC: myeloid-derived suppressor cell
TAM: tumor-associated macrophage
LOX-1: lectin-like oxidized low-density lipoprotein receptor 1
PD-1: programmed death 1
ROC: receiver operating characteristic (curve)
RFS: relapse-free survival
OS: overall survival
Treg: regulatory T cell
M-MDSC: monocytic MDSC
PMN-MDSC: polymorphonuclear MDSC
POD: post-operative day
NLR: neutrophil-to-lymphocyte ratio
PLR: platelet-to-lymphocyte ratio
CRP: C-reactive protein
