# Supplementary figures and images for: Involvement of CX3CR1+ cells appearing in the abdominal cavity in the immunosuppressive environment immediately after gastric cancer surgery
Source: World J Surg Oncol. 2024 Mar 4;22:74. doi: 10.1186/s12957-024-03353-1 (PMC10910822; doi:10.1186/s12957-024-03353-1)

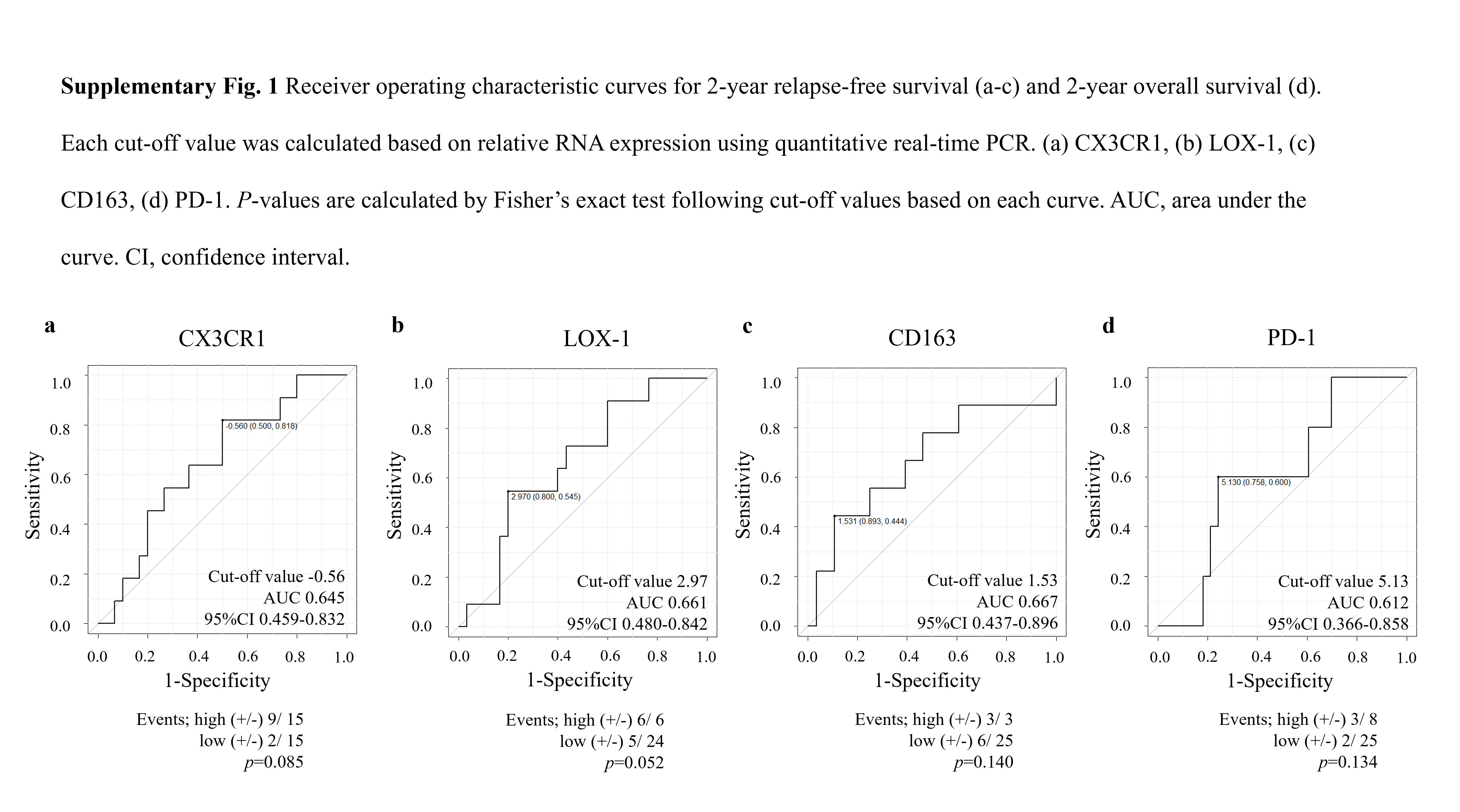

Supplement: Supplementary file 1 — Supplementary Material 1. [file 12957_2024_3353_MOESM1_ESM.tif]

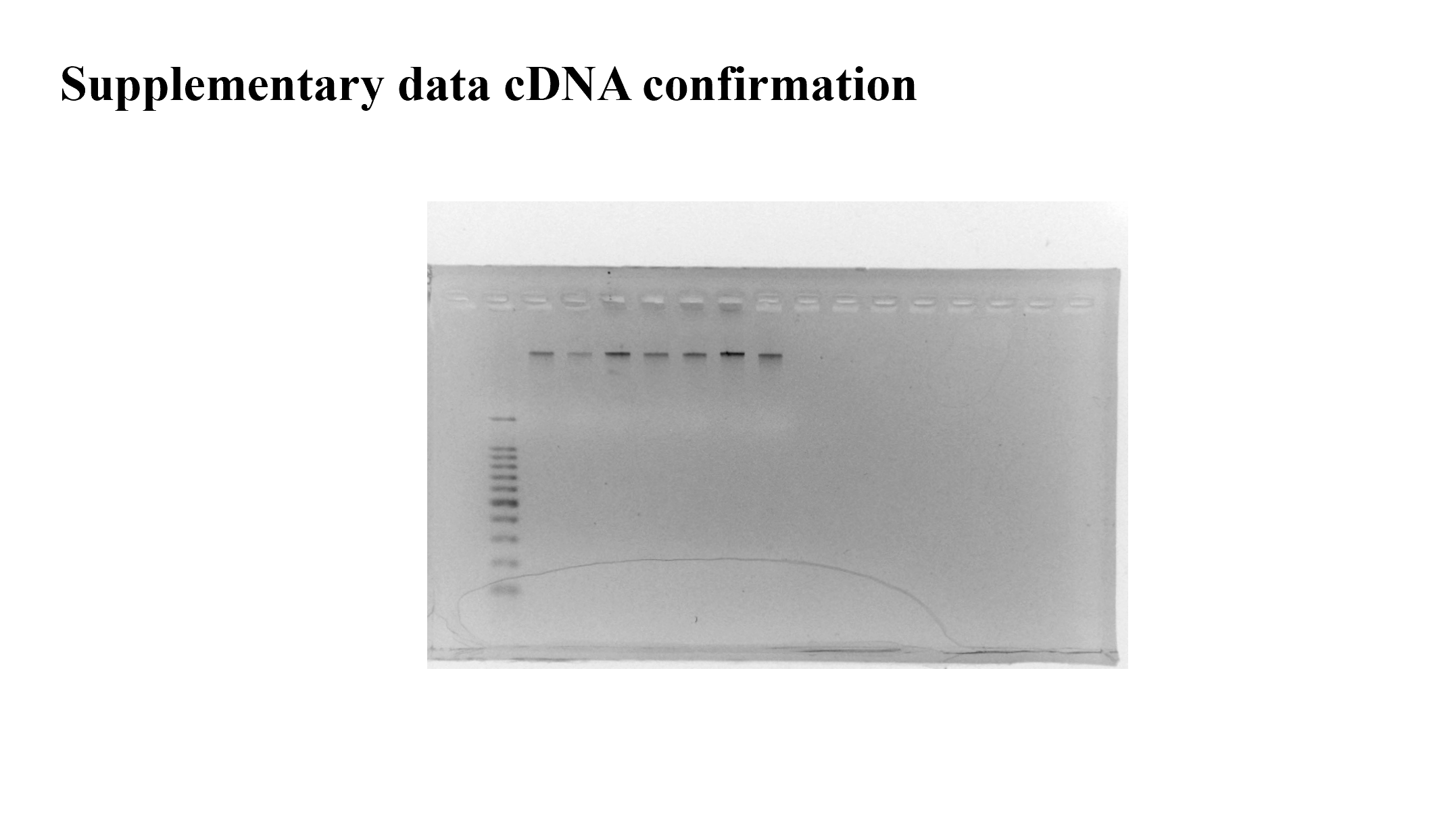

Supplement: Supplementary file 2 — Supplementary Material 2. [file 12957_2024_3353_MOESM2_ESM.tif]
